# 3β-Acet­oxy-8β,10β-dihy­droxy-6β-meth­oxy­eremophil-7(11)-en-8,12-olide

**DOI:** 10.1107/S1600536810029831

**Published:** 2010-07-31

**Authors:** Hai-Bo Wu, Hua Li, Xiao-Cong Lan, Wen-Shu Wang

**Affiliations:** a College of Life and Environment Science, Minzu University of China, Beijing 100081, People’s Republic of China

## Abstract

The title compound, C_18_H_26_O_7_, is an eremophilenolide which has been isolated from the plant *Ligularia duciformis* for the first time. The present study confirms the atomic connectivity assigned on the basis of ^1^H and ^13^C NMR spectroscopy. The mol­ecule contains three fused rings, two six-membered rings in chair confomations and a five-membered ring in a flattened envelope conformation. Two hy­droxy groups are involved in formation of intra- and inter­molecular O—H⋯O hydrogen bonds. The latter ones link mol­ecules into chains propagating in [010].

## Related literature

For related compounds extracted from *Ligularia duciformis* and *Cacalia roborowski*, see Wang *et al.* (2008 ▶) and Zhang *et al.* (1998 ▶), respectively.
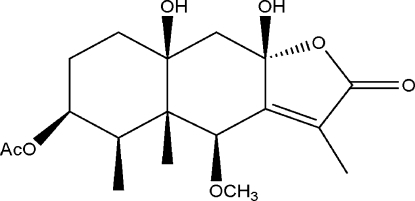


## Experimental

### 

#### Crystal data

C_18_H_26_O_7_
*M* *_r_* = 354.39
Orthorhombic, 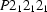

*a* = 7.3036 (15) Å
*b* = 9.789 (2) Å
*c* = 25.553 (5) Å
*V* = 1826.9 (6) Å^3^
*Z* = 4
Mo *K*α radiation
μ = 0.10 mm^−1^
*T* = 293 K
0.50 × 0.50 × 0.20 mm

#### Data collection

Rigaku R-AXIS RAPID IP diffractometer
Absorption correction: multi-scan (*ABSCOR*; Higashi, 1995 ▶) *T*_min_ = 0.952, *T*_max_ = 0.980
12058 measured reflections
1885 independent reflections
1203 reflections with *I* > 2σ(*I*)
*R*_int_ = 0.061

#### Refinement

*R*[*F* ^2^ > 2σ(*F* ^2^)] = 0.049
*wR*(*F* ^2^) = 0.139
*S* = 0.89
1885 reflections
227 parameters
H-atom parameters constrained
Δρ_max_ = 0.29 e Å^−3^
Δρ_min_ = −0.36 e Å^−3^

### 

Data collection: *RAPID-AUTO* (Rigaku, 2000 ▶); cell refinement: *RAPID-AUTO*; data reduction: *CrystalStructure* (Rigaku/MSC, 2000 ▶); program(s) used to solve structure: *SHELXS97* (Sheldrick,2008 ▶); program(s) used to refine structure: *SHELXL97* (Sheldrick, 2008 ▶); molecular graphics: *SHELXTL* (Sheldrick, 2008 ▶); software used to prepare material for publication: *SHELXL97*.

## Supplementary Material

Crystal structure: contains datablocks I, global. DOI: 10.1107/S1600536810029831/cv2743sup1.cif

Structure factors: contains datablocks I. DOI: 10.1107/S1600536810029831/cv2743Isup2.hkl

Additional supplementary materials:  crystallographic information; 3D view; checkCIF report

## Figures and Tables

**Table 1:** Hydrogen-bond geometry (Å, °)

*D*—H⋯*A*	*D*—H	H⋯*A*	*D*⋯*A*	*D*—H⋯*A*
O2—H2*A*⋯O3	0.82	2.19	2.879 (4)	142
O3—H3*A*⋯O2^i^	0.82	2.06	2.755 (4)	143

Symmetry code: (i) 
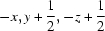
.

**Fig. 1.**
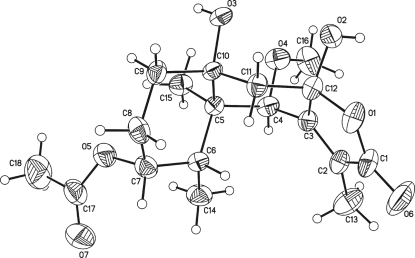
View of the title molecule showing the atom-numbering scheme. Displacement ellipsoids are drawn at the 50% probability level.

## supplementary crystallographic information

### Comment

*Ligularia duciformis*(C. Winkl.) Hand.-Mazz. grows in the western area of mainland China. It is used in traditional Chinese medicine having effects on dispelling phlegm and relieving cough activities. As a part of our research on biological resource by ethnic minorities in China, the title compound was isolated. Its structure was identified by NMR spectra data and compared with the previous reports  (Zhang *et al.*, 1998; Wang *et al.*, 2008). Herewith we present its crystal structure.

The molecule of the title compound contains a three-ring system A/B/C(Fig.1). There is a *cis* junction between the rings A(C9–C5/C10) and  B (C5–C3/C12–C10), while the methoxy group at C4 site and the hydroxyl group at C12 site are in the same orientation with two methyl groups at C6 and C5 sites. Rings A and B are both in chair conformations. The furan ring C (C12–C3/C2–C1/O1), of course, is in envelope-like conformations. Two hydroxy groups are involved in formation of intra- and intermolecular O—H···O hydrogen bonds (Table 1). The latter ones link molecules into chains propagated in direction [010].

### Experimental

The air-dried whole plant of *Ligularia duciformis* (1.5 kg) were pulverized and extracted three times with MeOH (each for 7 days) at room temperature. The extract was concentrated to give a residue (110 g), which was further separated by CC (SiO_2_, 200–300mesh, petroleum ether/EtOAc (30:1, 20:1, 15:1, 10:1, 8:1, 5:1, 3:1, 2:1, 1:1, 1:1.5 (*v*/*v*)) to yield 10 fractions: Fr. 1–10. Each fraction was examined by TLC and combined to afford many subfractions. Fr.8a (1.4 g) was subjected to CC (SiO_2_, 200–300mesh, petroleum ether/ EtOAc 8:1, 5:1 (*v*/*v*)) to provide the title compound (80 mg). ^1^H and ^13^C NMR spectral data of this compounds was recorded on Bruker-AV-500 s pectrometer, using CDCl_3_ as solvent and Me_4_Si as internal standard. The stereochemistry can be observed by X-ray diffraction experiment.

### Refinement

The hydrogen atoms were placed in calculated positions with C—H = 0.98-1.00 Å and O—H = 0.82 Å, and refined as riding with *U*_iso_(H) = 1.2-1.5Ueq(C,*O*). In the absence of any significant anomalous scatterers in the molecule, the absolute configuration has been arbitrarily assigned.

### Crystal data

**Table:**

C_18_H_26_O_7_	*F*(000) = 760
*M**_r_* = 354.39	*D*_x_ = 1.288 Mg m^−^^3^
Orthorhombic, *P*2_1_2_1_2_1_	Mo *K*α radiation, λ = 0.71073 Å
Hall symbol: P 2ac 2ab	Cell parameters from 12058 reflections
*a* = 7.3036 (15) Å	θ = 2.2–25.0°
*b* = 9.789 (2) Å	µ = 0.10 mm^−^^1^
*c* = 25.553 (5) Å	*T* = 293 K
*V* = 1826.9 (6) Å^3^	Block, colourless
*Z* = 4	0.50 × 0.50 × 0.20 mm

### Data collection

**Table:**

Rigaku R-AXIS RAPID IP diffractometer	1885 independent reflections
Radiation source: fine-focus sealed tube	1203 reflections with *I* > 2σ(*I*)
graphite	*R*_int_ = 0.061
Ω scans	θ_max_ = 25.0°, θ_min_ = 1.6°
Absorption correction: multi-scan (*ABSCOR*; Higashi, 1995)	*h* = 0→8
*T*_min_ = 0.952, *T*_max_ = 0.980	*k* = 0→11
12058 measured reflections	*l* = 0→30

### Refinement

**Table:**

Refinement on *F*^2^	Secondary atom site location: difference Fourier map
Least-squares matrix: full	Hydrogen site location: inferred from neighbouring sites
*R*[*F*^2^ > 2σ(*F*^2^)] = 0.049	H-atom parameters constrained
*wR*(*F*^2^) = 0.139	*w* = 1/[σ^2^(*F*_o_^2^) + (0.0856*P*)^2^] where *P* = (*F*_o_^2^ + 2*F*_c_^2^)/3
*S* = 0.89	(Δ/σ)_max_ < 0.001
1885 reflections	Δρ_max_ = 0.29 e Å^−^^3^
227 parameters	Δρ_min_ = −0.36 e Å^−^^3^
0 restraints	Extinction correction: *SHELXL97* (Sheldrick, 2008), Fc^*^=kFc[1+0.001xFc^2^λ^3^/sin(2θ)]^-1/4^
Primary atom site location: structure-invariant direct methods	Extinction coefficient: 0.130 (8)

### Special details

**Table:**

Experimental. Since the two skeleton methyl group in eremophilenolides are biogenic b position, we draw the relative stereochemistry of the title eremophilenolide, by reference to the structures of related eremophilenolides in Wang at al. (2008) and Zhang,*et al.* (1998) although the absolute configuration could not be reliably determined from anomalous dispersion effects. Furthermore, the relative stereochemistry in the title compound was confirmed by NMR data. ^13^C NMR (125 MHz, CDCl_3_, δ, p.p.m.): 177.0(C17), 170.2(C1), 152.9(C3), 129.7(C2), 103.4(C12), 79.8(C4), 74.7(C10), 71.4(C7), 57.9(C16), 47.4(C5), 44.3(C11), 36.5(C6), 30.0(C8), 27.5(C9), 21.3(C18), 12.8(C14), 12.5(C15), 8.8(C13).
Geometry. All e.s.d.'s (except the e.s.d. in the dihedral angle between two l.s. planes) are estimated using the full covariance matrix. The cell e.s.d.'s are taken into account individually in the estimation of e.s.d.'s in distances, angles and torsion angles; correlations between e.s.d.'s in cell parameters are only used when they are defined by crystal symmetry. An approximate (isotropic) treatment of cell e.s.d.'s is used for estimating e.s.d.'s involving l.s. planes.
Refinement. Refinement of *F*^2^ against ALL reflections. The weighted *R*-factor *wR* and goodness of fit *S* are based on *F*^2^, conventional *R*-factors *R* are based on *F*, with *F* set to zero for negative *F*^2^. The threshold expression of *F*^2^ > σ(*F*^2^) is used only for calculating *R*-factors(gt) *etc*. and is not relevant to the choice of reflections for refinement. *R*-factors based on *F*^2^ are statistically about twice as large as those based on *F*, and *R*- factors based on ALL data will be even larger.

### Fractional atomic coordinates and isotropic or equivalent isotropic displacement parameters (Å^2^)

**Table:**

	*x*	*y*	*z*	*U*_iso_*/*U*_eq_	
O1	−0.1967 (4)	−0.0086 (3)	0.16487 (12)	0.0560 (9)	
O2	−0.1302 (4)	0.1257 (3)	0.23691 (10)	0.0499 (8)	
H2A	−0.1081	0.2051	0.2450	0.075*	
O3	−0.0053 (4)	0.3976 (2)	0.21312 (9)	0.0431 (7)	
H3A	0.0292	0.4773	0.2139	0.065*	
O4	0.2582 (4)	0.2059 (3)	0.23680 (10)	0.0500 (8)	
O5	0.3015 (4)	0.4876 (3)	0.05334 (11)	0.0554 (9)	
O6	−0.1385 (6)	−0.2075 (3)	0.12421 (14)	0.0870 (13)	
O7	0.4013 (7)	0.4379 (4)	−0.02711 (15)	0.0977 (14)	
C1	−0.0818 (8)	−0.1056 (4)	0.14538 (19)	0.0605 (14)	
C2	0.1101 (7)	−0.0616 (4)	0.15266 (18)	0.0525 (12)	
C3	0.1075 (6)	0.0623 (4)	0.17441 (15)	0.0377 (10)	
C4	0.2533 (6)	0.1666 (4)	0.18310 (14)	0.0385 (9)	
H4A	0.3748	0.1278	0.1728	0.046*	
C5	0.2130 (5)	0.2971 (4)	0.14977 (15)	0.0331 (9)	
C6	0.2322 (6)	0.2612 (4)	0.09058 (14)	0.0400 (10)	
H6A	0.1475	0.1825	0.0844	0.048*	
C7	0.1687 (6)	0.3740 (4)	0.05353 (15)	0.0463 (11)	
H7A	0.1630	0.3351	0.0174	0.056*	
C8	−0.0162 (6)	0.4317 (5)	0.06655 (15)	0.0482 (11)	
H8A	−0.1115	0.3645	0.0567	0.058*	
H8B	−0.0365	0.5153	0.0456	0.058*	
C9	−0.0369 (6)	0.4666 (3)	0.12460 (15)	0.0408 (10)	
H9A	0.0431	0.5451	0.1331	0.049*	
H9B	−0.1650	0.4942	0.1315	0.049*	
C10	0.0124 (5)	0.3469 (3)	0.16006 (14)	0.0322 (9)	
C11	−0.1312 (6)	0.2326 (3)	0.15316 (15)	0.0374 (9)	
H11A	−0.2525	0.2676	0.1641	0.045*	
H11B	−0.1392	0.2083	0.1156	0.045*	
C12	−0.0877 (6)	0.1053 (4)	0.18429 (16)	0.0409 (10)	
C13	0.2662 (9)	−0.1493 (5)	0.1353 (2)	0.0872 (19)	
H13A	0.3821	−0.1035	0.1434	0.131*	
H13B	0.2578	−0.1648	0.0975	0.131*	
H13C	0.2611	−0.2371	0.1537	0.131*	
C14	0.4250 (6)	0.2095 (5)	0.07529 (16)	0.0563 (12)	
H14A	0.4278	0.1888	0.0378	0.084*	
H14B	0.4536	0.1266	0.0952	0.084*	
H14C	0.5159	0.2802	0.0832	0.084*	
C15	0.3518 (6)	0.4075 (4)	0.16654 (16)	0.0459 (11)	
H15A	0.4765	0.3748	0.1599	0.069*	
H15B	0.3372	0.4267	0.2040	0.069*	
H15C	0.3299	0.4912	0.1465	0.069*	
C16	0.3729 (6)	0.1197 (5)	0.26786 (17)	0.0645 (13)	
H16A	0.3710	0.1513	0.3043	0.097*	
H16B	0.4985	0.1233	0.2545	0.097*	
H16C	0.3277	0.0256	0.2662	0.097*	
C17	0.4023 (8)	0.5098 (6)	0.0103 (2)	0.0668 (14)	
C18	0.5228 (10)	0.6339 (6)	0.0173 (2)	0.099 (2)	
H18A	0.5956	0.6483	−0.0145	0.149*	
H18B	0.6048	0.6196	0.0471	0.149*	
H18C	0.4462	0.7144	0.0237	0.149*	

### Atomic displacement parameters (Å^2^)

**Table:**

	*U*^11^	*U*^22^	*U*^33^	*U*^12^	*U*^13^	*U*^23^
O1	0.058 (2)	0.0380 (14)	0.072 (2)	−0.0132 (17)	−0.0068 (17)	0.0012 (15)
O2	0.0555 (19)	0.0408 (14)	0.0533 (17)	0.0006 (15)	0.0123 (15)	0.0028 (14)
O3	0.0529 (17)	0.0325 (13)	0.0438 (14)	0.0023 (15)	0.0055 (15)	−0.0100 (12)
O4	0.0542 (19)	0.0575 (16)	0.0385 (15)	0.0164 (17)	−0.0133 (15)	−0.0020 (14)
O5	0.065 (2)	0.0504 (16)	0.0505 (17)	−0.0109 (17)	0.0103 (16)	0.0007 (14)
O6	0.123 (3)	0.0371 (17)	0.101 (3)	−0.020 (2)	−0.010 (3)	−0.0107 (18)
O7	0.136 (4)	0.086 (3)	0.071 (2)	−0.007 (3)	0.051 (3)	−0.005 (2)
C1	0.084 (4)	0.027 (2)	0.070 (3)	−0.005 (3)	0.001 (3)	0.003 (2)
C2	0.072 (3)	0.029 (2)	0.057 (3)	0.010 (2)	0.003 (3)	0.006 (2)
C3	0.044 (2)	0.029 (2)	0.040 (2)	0.011 (2)	−0.003 (2)	0.0035 (17)
C4	0.040 (2)	0.038 (2)	0.037 (2)	0.011 (2)	−0.009 (2)	−0.0018 (17)
C5	0.030 (2)	0.0316 (17)	0.038 (2)	−0.0008 (17)	−0.0052 (19)	−0.0009 (17)
C6	0.041 (2)	0.039 (2)	0.040 (2)	0.000 (2)	−0.001 (2)	−0.0067 (17)
C7	0.051 (3)	0.050 (2)	0.038 (2)	−0.001 (2)	−0.003 (2)	0.0031 (19)
C8	0.049 (3)	0.054 (2)	0.042 (2)	0.004 (2)	−0.003 (2)	0.015 (2)
C9	0.043 (2)	0.031 (2)	0.049 (2)	0.0072 (18)	0.000 (2)	0.0053 (17)
C10	0.036 (2)	0.0272 (18)	0.0331 (19)	0.0036 (18)	−0.0028 (19)	−0.0041 (16)
C11	0.033 (2)	0.0353 (19)	0.044 (2)	0.0033 (19)	−0.001 (2)	0.0067 (18)
C12	0.044 (2)	0.032 (2)	0.046 (2)	−0.004 (2)	−0.002 (2)	0.0001 (18)
C13	0.100 (5)	0.048 (3)	0.113 (5)	0.029 (3)	0.011 (4)	−0.019 (3)
C14	0.053 (3)	0.063 (3)	0.053 (3)	0.014 (2)	0.007 (2)	−0.007 (2)
C15	0.040 (2)	0.048 (2)	0.050 (2)	−0.004 (2)	−0.007 (2)	−0.010 (2)
C16	0.049 (3)	0.083 (3)	0.061 (3)	0.007 (3)	−0.017 (2)	0.024 (3)
C17	0.075 (4)	0.068 (3)	0.058 (3)	0.007 (3)	0.021 (3)	0.015 (3)
C18	0.100 (5)	0.085 (4)	0.113 (5)	−0.021 (4)	0.035 (4)	0.019 (4)

### Geometric parameters (Å, °)

**Table:**

O1—C1	1.361 (6)	C8—C9	1.530 (5)
O1—C12	1.458 (5)	C8—H8A	0.9900
O2—C12	1.394 (5)	C8—H8B	0.9900
O2—H2A	0.8200	C9—C10	1.524 (5)
O3—C10	1.450 (4)	C9—H9A	0.9900
O3—H3A	0.8200	C9—H9B	0.9900
O4—C4	1.425 (4)	C10—C11	1.544 (5)
O4—C16	1.429 (5)	C11—C12	1.512 (5)
O5—C17	1.342 (5)	C11—H11A	0.9900
O5—C7	1.476 (5)	C11—H11B	0.9900
O6—C1	1.208 (5)	C13—H13A	0.9800
O7—C17	1.186 (6)	C13—H13B	0.9800
C1—C2	1.478 (7)	C13—H13C	0.9800
C2—C3	1.334 (5)	C14—H14A	0.9800
C2—C13	1.494 (7)	C14—H14B	0.9800
C3—C4	1.492 (5)	C14—H14C	0.9800
C3—C12	1.508 (6)	C15—H15A	0.9800
C4—C5	1.563 (5)	C15—H15B	0.9800
C4—H4A	1.0000	C15—H15C	0.9800
C5—C15	1.543 (5)	C16—H16A	0.9800
C5—C6	1.559 (5)	C16—H16B	0.9800
C5—C10	1.566 (5)	C16—H16C	0.9800
C6—C7	1.526 (5)	C17—C18	1.511 (8)
C6—C14	1.547 (6)	C18—H18A	0.9800
C6—H6A	1.0000	C18—H18B	0.9800
C7—C8	1.501 (6)	C18—H18C	0.9800
C7—H7A	1.0000		
			
C1—O1—C12	108.7 (3)	O3—C10—C11	107.1 (3)
C12—O2—H2A	109.5	C9—C10—C11	109.2 (3)
C10—O3—H3A	108.8	O3—C10—C5	110.3 (3)
C4—O4—C16	113.0 (3)	C9—C10—C5	111.1 (3)
C17—O5—C7	119.0 (4)	C11—C10—C5	113.0 (3)
O6—C1—O1	121.9 (5)	C12—C11—C10	113.2 (3)
O6—C1—C2	128.5 (5)	C12—C11—H11A	108.9
O1—C1—C2	109.6 (4)	C10—C11—H11A	108.9
C3—C2—C1	107.7 (4)	C12—C11—H11B	108.9
C3—C2—C13	131.0 (5)	C10—C11—H11B	108.9
C1—C2—C13	121.3 (4)	H11A—C11—H11B	107.7
C2—C3—C4	132.4 (4)	O2—C12—O1	108.4 (3)
C2—C3—C12	109.7 (4)	O2—C12—C3	114.3 (3)
C4—C3—C12	117.3 (3)	O1—C12—C3	104.2 (3)
O4—C4—C3	110.2 (3)	O2—C12—C11	110.0 (3)
O4—C4—C5	108.0 (3)	O1—C12—C11	109.7 (3)
C3—C4—C5	110.1 (3)	C3—C12—C11	109.9 (3)
O4—C4—H4A	109.5	C2—C13—H13A	109.5
C3—C4—H4A	109.5	C2—C13—H13B	109.5
C5—C4—H4A	109.5	H13A—C13—H13B	109.5
C15—C5—C6	111.6 (3)	C2—C13—H13C	109.5
C15—C5—C4	107.3 (3)	H13A—C13—H13C	109.5
C6—C5—C4	109.1 (3)	H13B—C13—H13C	109.5
C15—C5—C10	110.5 (3)	C6—C14—H14A	109.5
C6—C5—C10	108.5 (3)	C6—C14—H14B	109.5
C4—C5—C10	109.8 (3)	H14A—C14—H14B	109.5
C7—C6—C14	110.9 (3)	C6—C14—H14C	109.5
C7—C6—C5	114.3 (3)	H14A—C14—H14C	109.5
C14—C6—C5	113.6 (3)	H14B—C14—H14C	109.5
C7—C6—H6A	105.7	C5—C15—H15A	109.5
C14—C6—H6A	105.7	C5—C15—H15B	109.5
C5—C6—H6A	105.7	H15A—C15—H15B	109.5
O5—C7—C8	108.0 (3)	C5—C15—H15C	109.5
O5—C7—C6	110.3 (3)	H15A—C15—H15C	109.5
C8—C7—C6	114.1 (3)	H15B—C15—H15C	109.5
O5—C7—H7A	108.1	O4—C16—H16A	109.5
C8—C7—H7A	108.1	O4—C16—H16B	109.5
C6—C7—H7A	108.1	H16A—C16—H16B	109.5
C7—C8—C9	112.8 (3)	O4—C16—H16C	109.5
C7—C8—H8A	109.0	H16A—C16—H16C	109.5
C9—C8—H8A	109.0	H16B—C16—H16C	109.5
C7—C8—H8B	109.0	O7—C17—O5	124.1 (5)
C9—C8—H8B	109.0	O7—C17—C18	125.2 (5)
H8A—C8—H8B	107.8	O5—C17—C18	110.6 (5)
C10—C9—C8	112.4 (3)	C17—C18—H18A	109.5
C10—C9—H9A	109.1	C17—C18—H18B	109.5
C8—C9—H9A	109.1	H18A—C18—H18B	109.5
C10—C9—H9B	109.1	C17—C18—H18C	109.5
C8—C9—H9B	109.1	H18A—C18—H18C	109.5
H9A—C9—H9B	107.9	H18B—C18—H18C	109.5
O3—C10—C9	105.8 (3)		
			
C12—O1—C1—O6	−175.9 (4)	C5—C6—C7—C8	−48.8 (5)
C12—O1—C1—C2	1.8 (5)	O5—C7—C8—C9	−75.5 (4)
O6—C1—C2—C3	175.2 (5)	C6—C7—C8—C9	47.6 (5)
O1—C1—C2—C3	−2.3 (5)	C7—C8—C9—C10	−52.6 (5)
O6—C1—C2—C13	−3.2 (8)	C8—C9—C10—O3	177.3 (3)
O1—C1—C2—C13	179.3 (4)	C8—C9—C10—C11	−67.7 (4)
C1—C2—C3—C4	−168.7 (4)	C8—C9—C10—C5	57.6 (4)
C13—C2—C3—C4	9.5 (8)	C15—C5—C10—O3	−50.2 (4)
C1—C2—C3—C12	1.8 (5)	C6—C5—C10—O3	−172.8 (3)
C13—C2—C3—C12	179.9 (5)	C4—C5—C10—O3	68.0 (3)
C16—O4—C4—C3	87.5 (4)	C15—C5—C10—C9	66.8 (4)
C16—O4—C4—C5	−152.2 (3)	C6—C5—C10—C9	−55.8 (4)
C2—C3—C4—O4	−126.6 (4)	C4—C5—C10—C9	−175.0 (3)
C12—C3—C4—O4	63.5 (4)	C15—C5—C10—C11	−170.1 (3)
C2—C3—C4—C5	114.4 (5)	C6—C5—C10—C11	67.3 (4)
C12—C3—C4—C5	−55.5 (4)	C4—C5—C10—C11	−51.9 (4)
O4—C4—C5—C15	51.6 (4)	O3—C10—C11—C12	−69.5 (4)
C3—C4—C5—C15	171.9 (3)	C9—C10—C11—C12	176.4 (3)
O4—C4—C5—C6	172.6 (3)	C5—C10—C11—C12	52.2 (4)
C3—C4—C5—C6	−67.0 (4)	C1—O1—C12—O2	−122.9 (4)
O4—C4—C5—C10	−68.6 (4)	C1—O1—C12—C3	−0.7 (4)
C3—C4—C5—C10	51.8 (4)	C1—O1—C12—C11	116.9 (4)
C15—C5—C6—C7	−70.3 (4)	C2—C3—C12—O2	117.5 (4)
C4—C5—C6—C7	171.4 (3)	C4—C3—C12—O2	−70.4 (4)
C10—C5—C6—C7	51.7 (4)	C2—C3—C12—O1	−0.7 (4)
C15—C5—C6—C14	58.4 (4)	C4—C3—C12—O1	171.4 (3)
C4—C5—C6—C14	−60.0 (4)	C2—C3—C12—C11	−118.2 (4)
C10—C5—C6—C14	−179.6 (3)	C4—C3—C12—C11	53.9 (4)
C17—O5—C7—C8	−124.0 (4)	C10—C11—C12—O2	76.8 (4)
C17—O5—C7—C6	110.8 (4)	C10—C11—C12—O1	−164.0 (3)
C14—C6—C7—O5	−57.0 (4)	C10—C11—C12—C3	−50.0 (4)
C5—C6—C7—O5	73.0 (4)	C7—O5—C17—O7	−5.4 (7)
C14—C6—C7—C8	−178.8 (3)	C7—O5—C17—C18	177.5 (4)

### Hydrogen-bond geometry (Å, °)

**Table:**

*D*—H···*A*	*D*—H	H···*A*	*D*···*A*	*D*—H···*A*
O2—H2A···O3	0.82	2.19	2.879 (4)	142
O3—H3A···O2^i^	0.82	2.06	2.755 (4)	143

Symmetry codes: (i) −*x*, *y*+1/2, −*z*+1/2.
